# Identification of differentially expressed proteins and clinicopathological significance of HMGB2 in cervical cancer

**DOI:** 10.1186/s12014-020-09308-4

**Published:** 2021-01-06

**Authors:** Xiao Han, Siyi Zhong, Pengnan Zhang, Yanmei Liu, Sangsang Shi, Congquan Wu, Shujun Gao

**Affiliations:** 1 Center of Diagnosis and Treatment for Cervical Diseases, Obstetrics and Gynecology Hospital of Fudan University, No. 419, Fangxie Road, Huangpu District, Shanghai, 200011 China; 2 Shanghai Key Laboratory of Female Reproductive Endocrine-Related Disease, Fudan University, Shanghai, 200011 China; 3 Department of Gynecology, Obstetrics and Gynecology Hospital of Fudan University, Shanghai, 200011 China

**Keywords:** Cervical cancer, Proteomics, HMGB2, Tumor progression

## Abstract

To investigate the complexity of proteomics in cervical cancer tissues, we used isobaric tags for relative and absolute quantitation (iTRAQ)-based mass spectrometry analysis on a panel of normal cervical tissues (N), high-grade squamous intraepithelial lesion tissues (HSIL) and cervical cancer tissues (CC). Total 72 differentially expressed proteins were identified both in CC vs N and CC vs HSIL. The expression of HMGB2 was markedly higher in CC than that in HSIL and N. High HMGB2 expression was significantly correlated with primary tumor size, invasion and tumor stage. The up-regulated HMGB2 was discovered to be associated with human cervical cancer. These findings suggest that HMGB2 may be a potentially prognostic biomarker and a target for the therapy of cervical cancer.

## Introduction

Cervical cancer is one of the most frequent malignant tumors and is the main cause of cancer-related mortality of women worldwide every year [1, 2]. Although the survival rates of the cancer patients have been improved, the advance in cervical cancer doesn’t match those observed in other common tumors, partly because of that cervical cancer patients were often diagnosed in an advanced stage [3]. Recently, the advances in biological behavior and molecular pathogenesis of cancers have resulted in the progression of molecular targeted therapies [4]. However, effective drug targets are limited. Thus, further research on molecular mechanisms underlying cervical cancer development is much needed to identify new therapeutic targets.

The molecular features of cancer are fiendishly complex because of cancer heterogeneity. As proteins are the primary functional macromolecule in organisms, proteomics attracted the public attention and are applied for various types of cancers. For example, by MS-based quantitative proteomic analysis, Moon-Won Yoo et al. identified and verified that four proteins were useful for discriminate patients with gastric cancer from normal persons [5]. Swiatly et al. highlighted several dysregulated proteins related to ovarian cancer by isobaric tags for relative and absolute quantification (iTRAQ)-based mass spectrometry analysis, in which five proteins were validated to differentially express in ovarian cancer tissues and could improve diagnostic performance [6]. For hepatocellular carcinoma research, 52 proteins were identified to interact with C reactive protein by iTRAQ-based proteomic profiling, contributing to the understanding of molecular pathogenesis of hepatocellular carcinoma [7]. All these researches showed the vital role of proteomics in the field of cancer investigation.

However, despite a proteomics analysis of serum samples from patients with cervical cancer and cervical intraepithelial neoplasis [8], there are few researches on dysregulated proteins analysis for cervical cancer tissue samples. Considering that several factors such as sample type and study design would affect the amount of proteomic studies data for cancer [9], we identified differentially expressed proteins in cervical cancer samples compared to high-grade squamous intraepithelial lesion samples and normal cervical samples by a particularly powerful tool iTRAQ-based mass spectrometry. Function annotation and protein–protein interaction network analysis of the identified proteins were carried out. Moreover, the highlighted protein was validated by immunohistochemistry staining; its clinical significance was assessed in cervical cancer patients.

## Materials and methods

### Patients and samples

Normal cervical tissues (N, n = 27), high-grade squamous intraepithelial lesion tissues (HSIL, n = 24) and cervical squamous cell cancer tissues (CC, n = 29) were obtained by colposcopy biopsy in the Obstetrics and Gynecology Hospital affiliated with Fudan University, China from 12/26/2014 to 12/25/2016. The HPV information of the samples was showed in Additional file 1: Table S1. The tumor stage of cervical cancer was categorized according to surgical and pathological findings, basing on the guidelines described by the International Federation of Gynecology and Obstetrics (FIGO) stage system. This research was approved by the review board and ethics committee of Obstetrics and Gynecology Hospital affiliated with Fudan University. Patients without any preoperative therapy were included. Written informed consents were obtained from all patients. For the iTRAQ-based mass spectrometry analysis, total 8 samples from CC, HSIL and N were selected, respectively. For the immunohistochemistry staining, all samples of the three groups were used.

### iTRAQ labeling, strongcatiobexchange fractionation and LC–MS/MS analysis

The protein concentration was detected with the BCA method. The iTRAQ labeling and strong catiob-exchange fractionation were performed according to the reported methods [10]. Firstly, 200 μg protein of each group was used for iTRAQ labeling, which was performed with tris (2-carboxyethyl) phosphine (TCEP) for 1 h of reduction at 60 °C and then with S-methyl methanethiosulfonate (MMTS) for 20 min of alkylation at room temperature. Subsequently, after 16 h of digestion at 37 °C using sequencing grade trypsin (Promega, San Luis Obispo, CA) (enzyme/protein (mass ration) = 1:20), the digestion production was separately labeled with 8-plex iTRAQ reagents (AB SCIEX, Washington, D.C.). After reconstituted with Solvent A (25% acetonitrile, 10 mM KH_2_PO_4_, pH 2.8), the samples were fractionated in the PolySULFOETHYL A column (200 × 4.6 mm, 5 μm, 200 Å, PolyLC Inc., Columbia, MD) with an Agilent 1260 series high performance liquid chromatography system. Peptides were fractionated more than 50 min with a constant velocity (350 μl/min) by a concentration gradient from 0 to 60% solvent B (10 mM, 25% acetonitrile and 350 mM KCl, pH 2.8). The fractions were collected and pooled into 20 fractions, followed by reconstituted with formic acid (0.1%) and desalted with C18 StageTips (3 M Empore, St. Paul, MN). LC-MS/MS analysis for the samples was performed on the LTQ-Orbitrap Velos mass spectrometer (Thermo Scientific, Bremen, Germany) with an instrument interface of Easy-nLC II system (Thermo Scientific, Bremen, Germany).

### Protein quantification and identification

Proteome Discoverer software (version 1.4.0.288; Thermo Fisher Scientific) was used to extract tandem mass spectra without performing charge state deconvolution and deisotoping. All MS/MS samples were analyzed with Mascot software (version 1.4.0.288; Matrix Science, London, UK) and Sequest software (version 1.4.0.288; Thermo Fisher Scientific, San Jose, CA, USA). Mascot and Sequest were separately set up to search SwissProt database (Homo sapiens, 20411 entries) with trypsin as the digestion enzyme. Mascot was searched with a fragment ion mass tolerance of 20 PPM and a parent ion tolerance of 10.0 PPM, while Sequest was searched with the same parent ion tolerance and different fragment ion mass tolerance (0.020 Da). Carbamidomethyl of cysteine and iTRAQ8plex of lysine and the n-terminus were specified as fixed modifications in both Mascot and Sequest. The variable modifications (oxidation of methionine and iTRAQ8plex of tyrosine) were also specified in both Mascot and Sequest. MS/MS based peptide and protein identifications were validated using Scaffold (version Scaffold_4.0.5, Proteome Software Inc., Portland, OR). If a probability of more than 99.0% to achieve a false discovery rate (FDR) less than 1.0% was established by the Scaffold Local FDR algorithm, the peptide identifications were accepted. While a probability of more than 99.0% to achieve a FDR less than 1.0%, the protein identifications were accepted. All the proteins identified contained at least 7 amino acids and at least 1 unique peptide. Protein Prophet algorithm was used to assign the protein probabilities. To meet the principles of parsimony, the proteins containing similar peptides, which couldn’t be differentiated by MS/MS analysis alone, were grouped. Matrix correction of channels in all samples were performed according to the reported i-Tracker algorithm [11]. The obtained intensity was globally normalized within all acquisition runs. Each quantitative sample was normalized in each acquisition run. The identification intensity of each peptide was normalized within the specified protein. The referenced channels were standardized to achieve a fold change of 1:1. All normalization computations used the medians to multiply and normalize the data. Mann Whitney Test analysis was performed to determine the differentially expressed proteins. Fold change (FC) of the proteins was calculated for the comparisons of CC vs N and CC vs HSIL, respectively. The proteins with a threshold of |–Log_2_FC|≥ 1.0 and *p* value < 0.05 were identified as significantly differentially expressed proteins.

### Bioinformatics analysis

The overlapped proteins of the significantly differentially expressed proteins in CC vs N and CC vs HSIL were selected for function annotation and protein–protein interaction (PPI) network analysis. The function annotation was carried out with DAVID Bioinformatics Resources (version 6.8; https://david.ncifcrf.gov/) [12, 13] and PANTHER Classification System (version 14.0; http://www.pantherdb.org/) [14], respectively. The PPI network analysis was carried out with STRING (version 11.0; https://string-db.org/) and the network was graphed with the Cytoscape software (version 3.6.0) [15, 16]. The sub-networks were identified using the MCODE plugin in Cytoscape with the default parameters.

### Immunohistochemistry staining

Paraffin-embedding tissues were cut to a thickness of 4 μm slides. After 10 min of antigen retrieval by the microwave oven at 95 °C, the slides were incubated with the primary antibody anti-HMGB2 (Ab124670, Abcam). The negative control slides were incubated with normal mouse IgG. The slides were rinsed with phosphate-buffered saline for several times and then stained with the Elivision TM Plus Polymer HRP (Mouse/Rabbit) IHC kit (Maixin Biological Technology Development Co., Fuzhou, China), which is based on the visualization of streptavidin–biotin-peroxidase. The slides were incubated with biotin-conjugated IgG (goat anti-mouse polyclonal antibody) for 20 min at 25 °C, and subsequently incubated with a streptavidin–biotin-HRP complex at 25 °C for 20 min. After washing, the slides were visualized via incubating with 3,3-diaminobenzidine solution (Maixin Biotechnology Development Co., Ltd). The nuclei were stained with hematoxylin.

### Assessment of immunohistochemical staining

The expression of HMGB2 expression was assessed semi-quantitatively based on immunohistochemical staining. The HMGB2 protein expression was separately evaluated by 2 blinded observers. Each slide obtained an immunoreactive score (IRS; 3, strong; 2, moderate; 1, weak; 0, negative) according to nuclear staining intensity. Cut point for high HMGB2 expression or positive: IRS ≥ 2; Cut point for low HMGB2 expression or negative: IRS < 2.

### Statistical analysis

SPSS (version 19.0, Chicago, IL, USA) was used for statistical analysis. Data were expressed as mean ± standard error of the mean (SEM). Pearson's chi-squared test and the Student two-sided t test were used to analyze the correlation between the expression of HMGB2 and clinicopathological factors. P < 0.05 was considered statistically significant.

## Results

### Identification of differentially expressed proteins

**Fig. 1:**
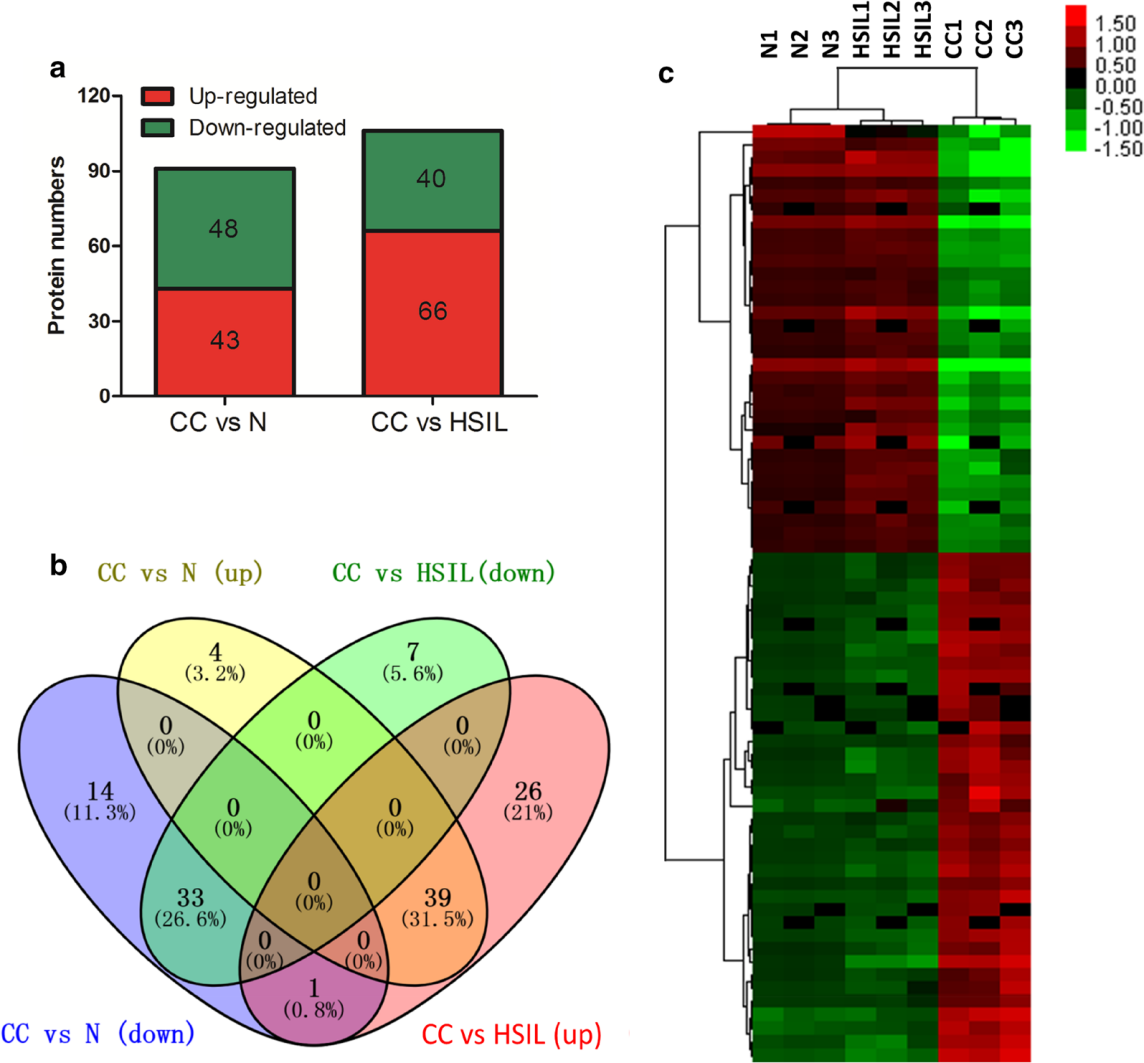
Identification of significantly differentially expressed proteins. **a** The protein numbers of significantly differentially expressed proteins in cervical cancer tissues (CC) compared to normal cervical tissues (N) and high-grade squamous intraepithelial lesion tissues (HSIL). **b** The overlapped proteins in the comparisons of CC vs N and CC vs HSIL. **c** The heatmap of overlap proteins. n = 3 indicate the three repeats from the pooled samples of each group

To characterize proteomic alterations in the primary cervical cancer samples, an iTRAQ-based mass spectrometry analysis approach was employed. Total 5473 proteins were identified in all the samples. Dysregulated proteins were quantified basing on the iTRAQ labels intensity for unique peptides. Total 91 significantly differentially expressed proteins were identified between cervical cancer samples and normal cervical samples (CC vs N); total 106 significantly differentially expressed proteins were identified between cervical cancer tissues and high-grade squamous intraepithelial lesion tissues (CC vs HSIL) (Fig. 1a). Among these proteins, 73 proteins were overlapped in the comparisons of CC vs N and CC vs HSIL (Fig. 1b). Of the 73 proteins, 72 proteins with the consistent regulatory relationships, namely simultaneous up-regulation or simultaneous down-regulation in CC vs N and CC vs HSIL, were selected for further analysis. The heatmap of 72 identified proteins suggested that three different groups (CC, N and HSIL) were distinguished by clustering (Fig. 1c).

### Function annotation of identified proteins

**Fig. 2:**
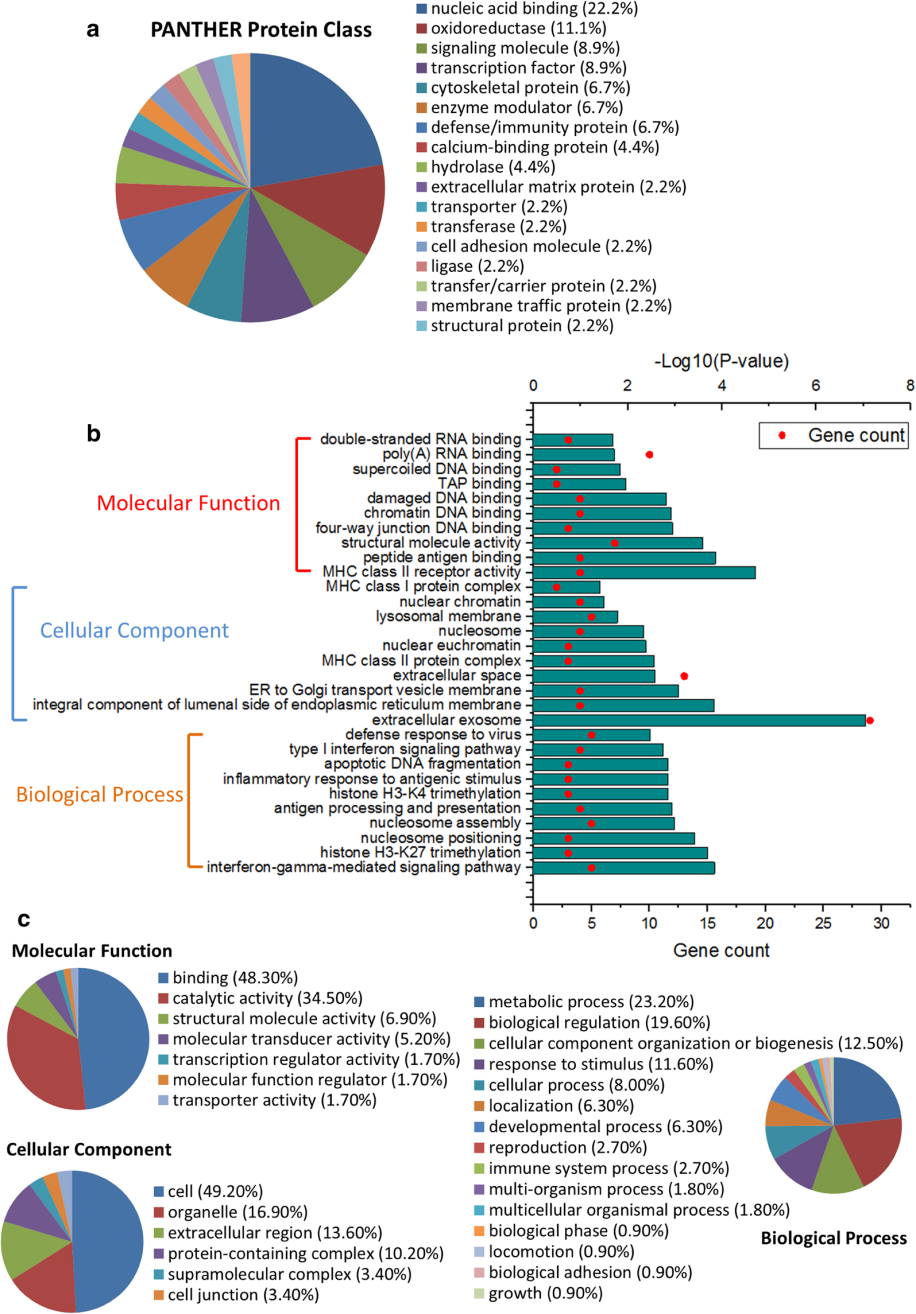
The function annotation of overlapped proteins was analyzed based on PANTHER Classification System and DAVID Bioinformatics Resources. **a** The PANTHER protein class of the overlapped proteins. The gene ontology analysis of overlapped proteins by DAVID (**b**) and PANTHER (**c**)

Based on the PANTHER Classification System, 72 identified proteins were categorized into 18 protein classes (Fig. 2a). Nucleic acid binding (22.2%), oxidoreductase (11.1%), signaling molecule (8.9%) and transcription factor (8.9%) were the four largest classes. Gene ontology (GO) analysis of 72 identified proteins was performed on both PANTHER and DAVID from three aspects: molecular function, cellular component and biological process. The top 10 enriched GO terms by DAVID were showed in Fig. 2b; the mainly enriched GO terms by PANTHER were showed in Fig. 2c. Both the GO results of PANTHER and DAVID showed that the identified proteins were mainly related to binding and structural molecule activity, involved in biological regulation and various immune-related biological process (antigen processing and presentation, inflammatory response to antigenic stimulus, defense response to virus, response to stimulus, immune system process, etc.), and were presented in extracellular region, membrane and protein complex. The top 10 GO terms (biological process and molecular function) and involved proteins analyzed by DAVID are shown in Table 1.

**Table 1:** Top 10 GO terms (biological process and molecular function) and involved proteins by DAVID

GO ID	Term	*P* value	Involved proteins	Fold Enrichment
Biological process				
GO:0060333	Interferon-gamma-mediated signaling pathway	1.45 × 10^–4^	HLA-DRB1, HLA-C, HLA-B, HLA-DRA, GBP1	18.48
GO:0098532	Histone H3-K27 trimethylation	2.06 × 10^−4^	HIST1H1E, HIST1H1D, HIST1H1C	131.19
GO:0016584	Nucleosome positioning	3.82 × 10^−4^	HIST1H1E, HIST1H1D, HIST1H1C	98.39
GO:0006334	Nucleosome assembly	1.03 × 10^−3^	H1F0, HIST1H1E, HMGB2, HIST1H1D, IST1H1C	11.02
GO:0019882	Antigen processing and presentation	1.14 × 10^−3^	HLA-DRB1, HLA-C, HLA-B, HLA-DRA	19.09
GO:0080182	Histone H3-K4 trimethylation	1.41 × 10^−3^	HIST1H1E, HIST1H1D, HIST1H1C	52.48
GO:0002437	Inflammatory response to antigenic stimulus	1.41 × 10^−3^	HMGB1, HMGB2, HLA-DRB1	52.48
GO:0006309	Apoptotic DNA fragmentation	1.41 × 10^−3^	H1F0, HMGB1, HMGB2	52.48
GO:0060337	Type I interferon signaling pathway	1.78 × 10^−3^	IFIT1, HLA-C, HLA-B, ISG20	16.40
GO:0051607	Defense response to virus	3.41 × 10^−3^	AZU1, IFIT1, DEFA3, ISG20, GBP1	7.96
Molecular function				
GO:0032395	MHC class II receptor activity	1.98 × 10^−5^	KRT17, HLA-DRB1, HLA-C, HLA-DRA	72.61
GO:0042605	Peptide antigen binding	1.38 × 10^−4^	HLA-DRB1, HLA-C, HLA-B, HLA-DRA	38.90
GO:0005198	Structural molecule activity	2.62 × 10^−3^	KRT6C, PGM5, KRT17, FLG, SPRR1B, KRT1, SPRR3	7.72
GO:0000400	Four-way junction DNA binding	1.14 × 10^−3^	HMGB1, MSH6, HMGB2	58.34
GO:0031490	Chromatin DNA binding	1.20 × 10^−3^	H1F0, HIST1H1E, HIST1H1D, HIST1H1C	18.78
GO:0003684	Damaged DNA binding	1.53 × 10^−3^	HMGB1, MSH6, HMGB2, MSH5	17.29
GO:0046977	TAP binding	1.08 × 10^−2^	HLA-C, HLA-B	181.52
GO:097100	Supercoiled DNA binding	1.44 × 10^−2^	HMGB1, HMGB2	136.14
GO:0044822	Poly(A) RNA binding	1.94 × 10^−2^	H1F0, HMGB1, HIST1H1E, HMGB2, HIST1H1D, HIST1H1C, TRA2B, RPL26, TMSB4X, MANF	2.41
GO:0003725	Double-stranded RNA binding	2.05 × 10^−2^	HMGB1, YRDC, TUBB4B	13.39

### PPI network analysis of identified proteins

**Fig. 3:**
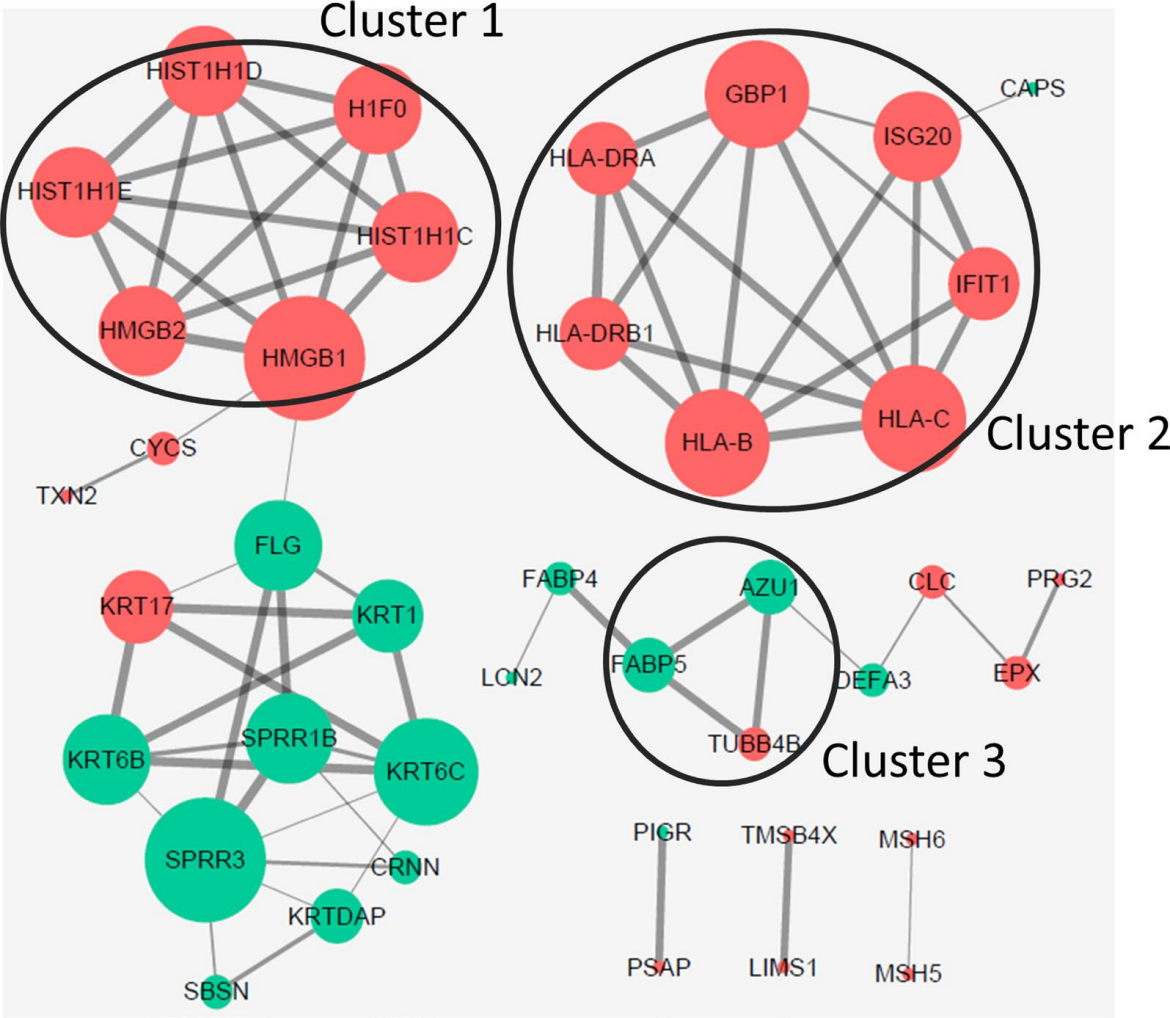
The protein–protein network analysis of overlapped proteins based on STRING online database. Cluster 1, 2 and 3: the sub-networks identified by MCODE plugin in the Cytoscape. The red and green indicate to significantly up-regulated and down-regulated proteins in cervical cancer samples compared to both high-grade squamous intraepithelial lesion samples and normal cervical samples, respectively

PPI network analysis was performed using STRING. Three clusters were identified by sub-networks analysis using MCODE plugin in the Cytoscape software (Fig. 3). The cluster 1 was mainly composed of HMG box transcription factor chromatin and chromatin-binding protein signaling molecule (HMGB1 and HMGB2), and histone (H1F0, HIST1H1C, HIST1H1D and HIST1H1E). The cluster 2 was made up of HLA-DRA, HLA-B, HLA-DRB1, HLA-C, GBP1, ISG20 and IFIT1. Among these proteins, HLA-DRA and HLA-DRB1 was belonged to major histocompatibility complex antigen protein class. The cluster 3 contained tubulin TUBB4B. Here, we reported that the up-regulated HMGB2 was associated with human cervical cancer. Thus, the expression of HMGB2 was validated and its clinical significance was studied further.

### Immunohistochemical expression of HMGB2

**Fig. 4:**
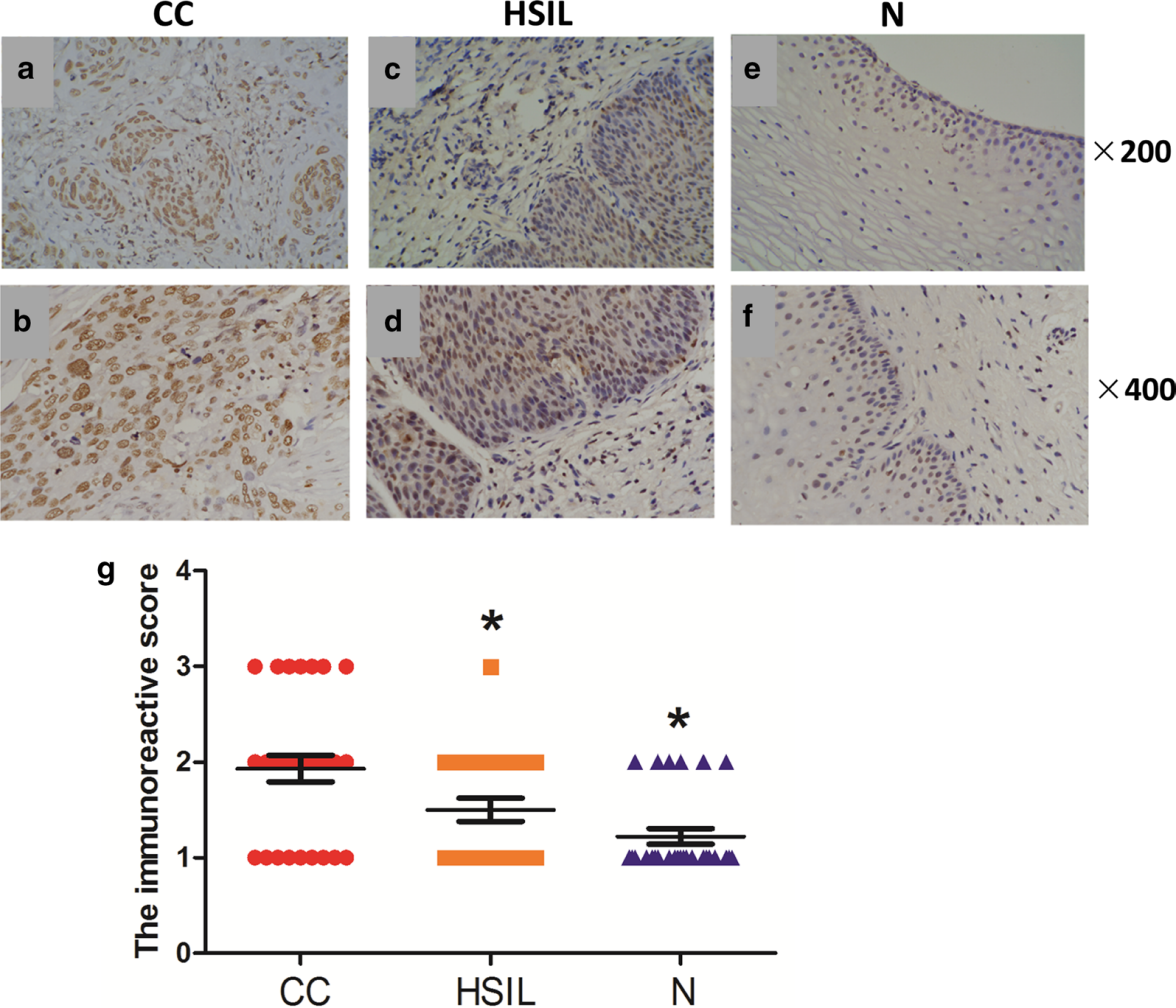
Immunohistochemical expression of HMGB2 in cervical cancer samples (CC), normal cervical samples (N) and high-grade squamous intraepithelial lesion samples (HSIL). **a**–**f** Representative immunohistochemical staining images for CC, N and HSIL. **g** The immunoreactive score for CC, N and HSIL. *P < 0 0.05 compared to CC

The expression of HMGB2 was determined by immunohistochemistry staining. There were no tissues represented negative immunohistochemical staining for HMGB2. The representative microphotographs of IRS score 1, 2 and 3 were shown in Additional file 1: Figure S1. As shown in Fig. 4a–f, immunohistochemistry stains of HMGB2 in almost all the samples were in nucleus. Strong HMGB2 immunoreactivity (20/29) was found in cervical cancer tissues (Fig. 4a, b). Most of high-grade squamous intraepithelial lesion tissues (HSIL) had weak nuclear stains for HMGB2 (Fig. 4c, d). Only 4 of 24 HSIL samples showed moderate immunoreactivity of HMGB2. Almost no cases showed HMGB2 immunoreactivity in those normal cervical tissues (Fig. 4e, f). Overall, the expression of HMGB2 in cervical cancer samples was significantly higher than in HSIL and normal cervical samples (both *P* < 0.05; Fig. 4g). Interestingly, positive reaction of HMGB2 was gradually higher during the progression of cervical cancer.

### HMGB2 expression in was associated with clinicopathological factors

To investigate the correlations between HMGB2 expression and clinicopathological factors in cervical cancer, the expression level of HMGB2 was represented with an immunoreactive scored (IRS; 3, strong; 2, moderate; 1, weak; 0, negative). Of 29 cervical cancer samples, 9 (35.5%) samples with IRS of 0-1 were classified as low HMGB2 expression (HMGB2-low), and 20 (64.5%) samples with IRS of 2-3 were classified as high HMGB2 expression (HMGB2-high). Table 2 showed the correlation analysis between HMGB2 expression and clinicopathological parameters of cervical cancer patients. The expression of HMGB2 was significantly associated with primary tumor size, invasion (infiltration depth) and FIGO stage (*P* < 0.05, respectively).

**Table 2:** Correlations of HMGB2 expression with clinicopathological parameters in primary cervical cancer tissues

Variables	No. of cases	HMGB2 expression		*P* value
		Low	High	
Age (year)				
< 50	15	4	11	0.273
≥ 50	14	5	9	
Family history of malignant tumors				
Yes	3	1	2	0.460
No	26	8	18	
P16 expression				
Positive	29	9	20	
Negative	0	0	0	
Primary tumor size(cm)				
< 3	7	5	2	0.015*
≥ 3	22	4	18	
Pelvic lymph node metastasis				
Positive	8	2	6	0.325
Negative	21	7	14	
Infiltration depth				
Superficial/Deep fibrous histiocytoma	18	8	10	0.048*
Outer layer of the cervix	11	1	10	
The FIGO stage				
Ia-Ib1	15	8	7	0.009*
Ib2-IIa2	14	1	13	

*P < 0.05

*FIGO* International Federation of Gynecology and Obstetrics

## Discussion

Dysregulated-proteins identification is of great significance for the discovery of biomarker, which can contribute to the early detection, therapeutic intervention and disease prognosis. In this study, total 72 differentially expressed proteins were identified to up-regulate in cervical cancer samples compared to both high-grade squamous intraepithelial lesion samples and normal cervical samples. Most of the 72 identified proteins were categorized into the nucleic acid binding (22.2%) of PANTHER protein class. Gene ontology (GO) analysis results revealed that identified proteins were mainly related to binding and structural molecule activity, involved in biological regulation and various immune-related biological process, and were presented in extracellular region, membrane and protein complex. The results of protein–protein interaction network analysis identified three clusters of highlighted proteins. Among these highlighted proteins, the members of HMG box transcription factor chromatin and chromatin-binding protein signaling molecule (HMGB1 and HMGB2) attracted our attention. Here, we reported that the up-regulated HMGB2 was associated with human cervical cancer.

As ubiquitous and abundant nuclear non-histone chromosomal proteins, high-mobility group box (HMGB) proteins play an important role in binding to distorted DNA structures and subsequently regulating its transcription, replication, repair and recombination [17]. The HMGB family is comprised with HMGB1, HMGB2, HMGB3 and HMGB4. HMGB1 and HMGB2, which have greater than 80% identity of amino acid, are highly conserved with indistinguishable biological properties, including binding to DNA without specificity of the sequence [18]. It has been reported that HMGB1 is associated with a variety of diseases, including sepsis, arthritis and cancer [19–21]. Overexpression of HMGB1 has been found in numerous human cancers, such as pancreatic cancer [22], prostate cancer [23], breast cancer [24, 25], melanoma [26], colorectal cancer [27] and leukemia [28]. More importantly, HMGB proteins preferred to bind to mis-incorporated nucleoside analogues or to cis-platinum (II) diamine dichloride (cisplatin)–modified DNA and subsequently inhibited the excision repair of nucleotide, which could have important value for cancer therapy [29–31]. Notably, HMGB1 expression has been reported to be related to tumor stage, invasion and metastasis in cervical squamous cell carcinoma as early as in year 2008 [32].

Considering the high homology of HMGB2 compared to HMGB1, it might have a similar role in the development of cancer. Koon et al. reported that HMGB2 was overexpressed in the malignant gastrointestinal stromal tumors and might be associated with the malignant behavior of gastrointestinal stromal tumors [33]. Then, overexpression of HMGB2 has been found in different kinds of human cancers, such as skin cancer [34], glioblastoma [35], hepatocellular carcinoma [36] and pancreatic cancer [37]. Lately, HMGB2 has been found to overexpress and promote cell proliferation and radiosensitivity through retinoblastoma-interaction-dependent or independent mechanisms [38]. Our recent research revealed that, according to the RNA-Seq data from The Cancer Genome Atlas (TCGA) program and The Genotype-Tissue Expression (GTEx) project, HMGB2 expression was significantly higher in cervical squamous cell carcinoma and endocervical adenocarcinoma than that in the normal controls; up-regulated HMGB2 expression promoted cell proliferation by activating AKT signaling pathway in cervical cancer cell lines [39]. Additionally, high expression of HMGB2 was associated with a poor prognosis for the patients with breast cancer via promoting cell proliferation and glycolysis in breast cancer cells [40, 41]. However, the expression and function of HMGB2, especially its relevance in carcinogenesis in cervical cancer remains largely unknown. Therefore, we detected the expression of HMGB2 in cervical cancer tissues by immunohistochemistry and evaluated the significance of HMGB2 expression in the clinical further. Here, our findings indicated that high expression of HMGB2 was significantly associated with primary tumor size, invasion (infiltration depth) and FIGO stage in cervical cancer. Although the exactly underlying mechanism of HMGB in prognosis of patients with cervical cancer remained uncovered, our results revealed that HMGB2 might at least partly promote cervical cancer progression and it might be a potentially prognostic biomarker for cervical cancer patients. These findings might facilitate future researches on the function of HMGB2 in cervical cancer.

Certainly, there are some limitations in this work. A limitation is that the small sample size. Eight samples of each group were randomly selected and then analyzed by iTRAQ 8 labeling and MS/MS. The way in which the samples were selected might resulted in some errors, and if the other different samples were selected, the estimation of FDR using sample permutation analysis might produce another different result. Notably, the result of immunohistochemical staining was consistent with that of proteomics analysis and thus enhanced the credibility. Of course, in view of total 53 patient samples (24 high-grade squamous intraepithelial lesion tissues and 29 cervical squamous cell cancer tissues), a large numbers of patients will contribute to understand the correlation between HMGB2 expression and clinicopathological parameters of cervical cancer patients better. Additionally, it should be noticed that the different cellularity of normal cervical tissues, high-grade squamous intraepithelial lesion tissues and cervical squamous cell cancer tissues might be an interference factor during the analysis.

In conclusion, notwithstanding these limitations, we identified 72 differentially expressed proteins in cervical cancer samples compared to normal cervical samples and high-grade squamous intraepithelial lesion samples. Most of the identified proteins were nucleic acid binding proteins. Moreover, further research results showed that HMGB2 was frequently up-regulated in cervical cancer samples and its up-regulation was associated with primary tumor size, infiltration depth and FIGO stage, resulting in tumor progression. These results suggest that HMGB2 may contribute to the progression of cervical cancer and that the presence of HMGB2 in cervical cancer tissues may be a prognostic indicator for cervical cancer patients.

## Supplementary Information

**Additional file 1: Table S1.** The HPV information of the samples. **Figure S1.** The representative microphotographs of IRS score 1, 2 and 3 for HMGB2.

## Data Availability

All data generated or analyzed during this study are included in this published article.
