# Supplementary material for: Identification of differentially expressed proteins and clinicopathological significance of HMGB2 in cervical cancer
Source: Clin Proteomics. 2021 Jan 6;18:2. doi: 10.1186/s12014-020-09308-4 (PMC7789524; doi:10.1186/s12014-020-09308-4)
Supplement: Supplementary file 1 — Additional file 1: Table S1. The HPV information of the samples. Figure S1. The representative microphotographs of IRS score 1, 2 and 3 for HMGB2. [file 12014_2020_9308_MOESM1_ESM.docx]

**Supplementary table 1** The HPV information of the samples

| Tissues | HPV | | | Total |
| --- | --- | --- | --- | --- |
|  | + | － | No result |  |
| N | 0 | 27 | 0 | 27 |
| HSIL | 22 | 2 | 0 | 24 |
| CC | 9 | 0 | 20^#^ | 29 |
| Total | 29 | 27 | 20 | 80 |

Note: N, normal cervical; HISL, high-grade squamous intraepithelial lesion; CC, cervical cancer. ^#^HPV test is mainly used in cervical cancer screen and follow up. Such patients were transferred from and had been diagnosed by other hospitals. We did not check their HPV.

**
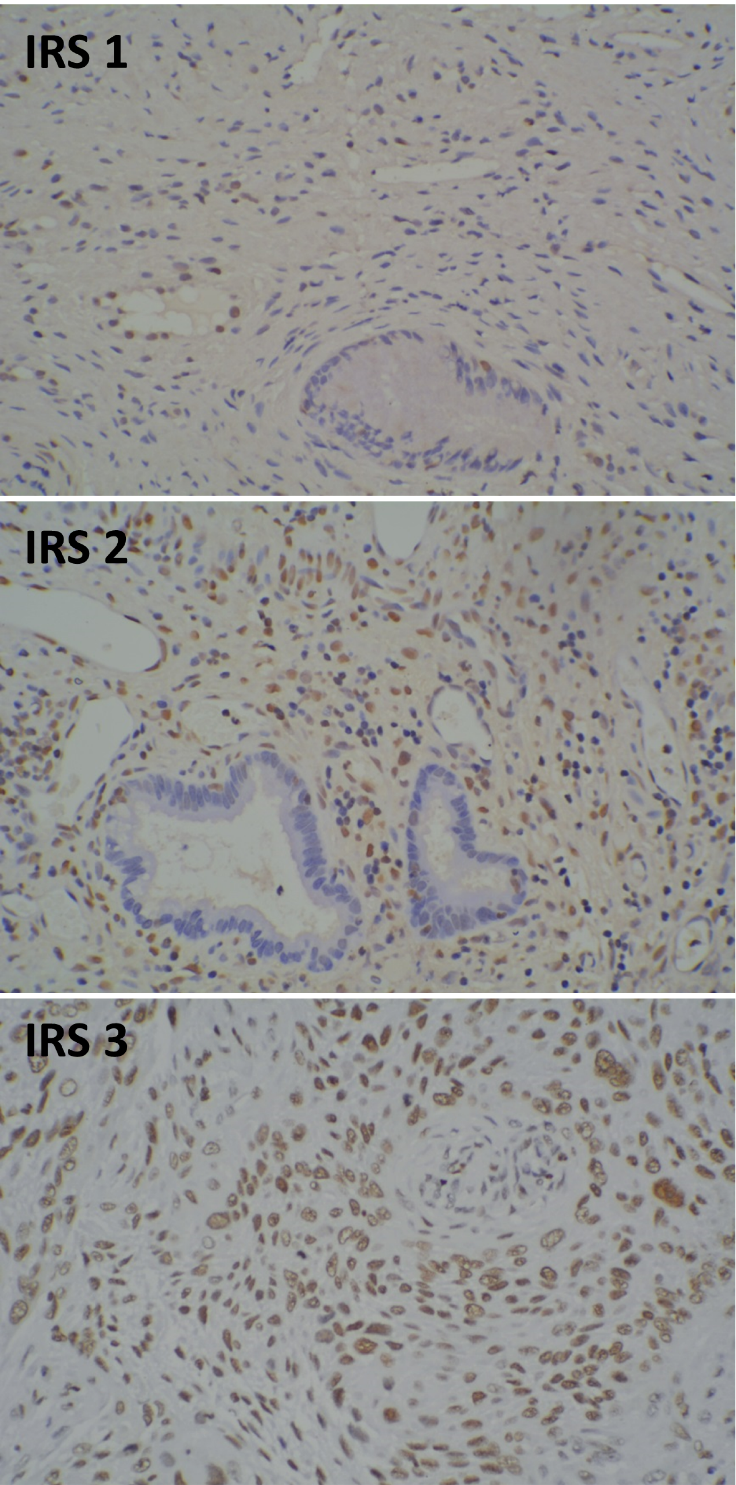
**

**Supplementary Figure S1** The representative microphotographs of IRS score 1, 2 and 3 for HMGB2
